# Inunction in Scarlet Fever

**Published:** 1893-09-02

**Authors:** 


### INUNCTION IN SCARLET FEVER.

Mr. Kingsett writes from the Sanitas Co.: With this letter I purpose closing my discussion with Dr. Curgenven, who concedes that I "may be a good chemist," but denies that I am a physiologist. I am content with the reputation he does not seek to destroy, and will only remind him that at least I am the author of a well-known scientific treatise on physiological chemistry, which was published in 1878 by Longmans and Co. As for the immediate subject under discussion, Dr. Curgenven has constantly shifted his ground, and now states that inunction of children with "Sanitas" oil would so obstruct the action of the skin that fatal results would follow. I reply that I have never recommended any such proposal, and only urge that if "Sanitas" oil be employed instead of the particular kind of eucalyptus oil, in which he appears to be so deeply interested for all purposes, it would serve infinitely better. Applied as an inunction with another suitable basis, "Sanitas" oil would not obstruct in any dangerous sense the action of the skin, and would do better service as an antiseptic and disinfectant. Dr. Curgenven may be a good medical man, but I have sufficient physiological knowledge to properly appreciate the absorptive capacity of children's skins. In conclusion, I may remind your readers that in the first place Dr. Curgenven claimed for some kind of eucalyptus oil superior properties to those of "Sanitas" oil as an antiseptic, and that I denied this claim and challenged him to substantiate it, which challenge he is evidently afraid of accepting, or, at least, has not accepted. I do not care in the least who makes the investigation, but I know that careful experiments will demonstrate that "Sanitas" oil, which contains 50 per cent. volatile constituents, is much more powerful as an antiseptic and disinfectant than any kind of eucalyptus oil, and that its proper employment in inunction will not be attended with any deleterious, much less fatal, results as suspiciously hinted at by Dr. Curgenven, whose suspicions seem to be as wild as his hypothetical statements concerning matters with which he has evidently a very poor acquaintance.

